# Anti-Inflammatory and Antioxidant Properties of a New Mixture of Vitamin C, Collagen Peptides, Resveratrol, and Astaxanthin in Tenocytes: Molecular Basis for Future Applications in Tendinopathies

**DOI:** 10.1155/2024/5273198

**Published:** 2024-07-30

**Authors:** Monica Marzagalli, Stefania Battaglia, Michela Raimondi, Fabrizio Fontana, Marco Cozzi, Francesca R. Ranieri, Roberto Sacchi, Valeria Curti, Patrizia Limonta

**Affiliations:** ^1^ Department of Pharmacological and Biomolecular Sciences “Rodolfo Paoletti” University of Milano, Milano 20133, Italy; ^2^ R&D Department Kolinpharma S.p.A., Lainate 20045, Italy; ^3^ Department of Earth and Environmental Sciences University of Pavia, Pavia 27100, Italy

## Abstract

Tendinopathy is one of the most frequent musculoskeletal disorders characterized by sustained tissue inflammation and oxidative stress, accompanied by extracellular matrix remodeling. Patients suffering from this pathology frequently experience pain, swelling, stiffness, and muscle weakness. Current pharmacological interventions are based on nonsteroidal anti-inflammatory drugs; however, the effectiveness of these strategies remains ambiguous. Accumulating evidence supports that oral supplementation of natural compounds can provide preventive, and possibly curative, effects. Vitamin C (Vit C), collagen peptides (Coll), resveratrol (Res), and astaxanthin (Asx) were reported to be endowed with potential beneficial effects based on their anti-inflammatory and antioxidant activities. Here, we analyzed the efficacy of a novel combination of these compounds (Mix) in counteracting proinflammatory (IL-1*β*) and prooxidant (H_2_O_2_) stimuli in human tenocytes. We demonstrated that Mix significantly impairs IL-6-induced IL-1*β* secretion, NF-*κ*B nuclear translocation, and MMP-2 production; notably, a synergistic effect of Mix over the single compounds could be observed. Moreover, Mix was able to significantly counteract H_2_O_2_-triggered ROS production. Together, these results point out that Mix, a novel combination of Vit C, Coll, Resv, and Asx, significantly impairs proinflammatory and prooxidant stimuli in tenocytes, mechanisms that contribute to the onset of tendinopathies.

## 1. Introduction

Musculoskeletal disorders (MSDs) are injuries or dysfunctions involving different parts of the musculoskeletal system, such as muscles, nerves, tendons, joints, cartilage, ligaments, and spinal discs.

Tendon is a dense connective tissue made of sparse specialized fibroblasts (named tenocytes) embedded in an abundant extracellular matrix mainly composed of collagen fibers, mostly type I collagen (95%) but also collagens III, V, and X; proteoglycans; and elastin. Tendon establishes connections between skeletal muscles and bones, thus transferring them the contraction forces to support the movements around a joint [1, 2, 3].

Tendinopathy is one of the most common MSDs which could affect individuals of different ages, sex, physical activity, and health condition [4].

Tendon degeneration is characterized by alterations of the tissue consisting of a peculiar remodeling of the extracellular matrix: fragmentation of collagen fibers, increased expression of matrix metalloproteinases (MMPs), accumulation of proteoglycans, increased neoinnervation and neovascularization. Most cases of tendinopathy are associated with pain, swelling, stiffness and reduced mobility at the affected joint, muscle weakness, and consequent decline in function [4, 5, 6].

Tendinopathy most frequently affects shoulders (rotator cuff tendons), elbows (extensor carpi radialis brevis tendon), hips (gluteal tendons), knees (patellar tendons), and feet or ankles (Achilles tendon and posterior tibial or peroneal tendons). The exact etiology of this disease is still unclear. However, different extrinsic and intrinsic factors were widely reported to be involved in its development. Among intrinsic factors, there are aging, metabolic disorders, such as obesity and diabetes mellitus, familial history and genetic factors, muscle weakness, altered tendon structure and homeostasis, and medications (hormone replacement therapy and fluoroquinolone antibiotics). Concerning extrinsic factors, tendon overuse could represent a risk factor that increases the prevalence and incidence in the population subjected to intense sport training or occupational tasks involving repetitive movements [4, 6, 7, 8, 9, 10].

Over the years, it has become increasingly clear that inflammation is deeply involved in the development and progression of tendinopathy as well as in tendon healing.

Specifically, it is now well recognized that tendon inflammation and degeneration are strictly interconnected, with inflammation preceding tissue alterations thus being the driver of the pathology [11, 12, 13, 14, 15]. Different immune cell subtypes and proinflammatory agents have been found to be implicated in these mechanisms. In physiological conditions, the transcription factor nuclear factor-*κ* B (NF-*κ*B) is kept in an inactive state by the specific inhibitor I*κ*B. It has been shown that inflammatory stimuli induce the recruitment of IKK (the I*κ*B kinase complex) leading to I*κ*B phosphorylation and degradation, thus allowing the nuclear translocation of NF-*κ*B [16, 17]. An increased expression of NF-*κ*B, as well as its accumulation at the nuclear level, was observed in tissue samples of rotator cuff in early-stage tendinopathies [15].

In the last decade, it has also been widely reported that tendon-infiltrating immune cells and tenocytes activate a vicious communication mediated by the secretion of a wide range of inflammatory chemokines and cytokines, responsible for the promotion of significant alterations in tendon structure and composition, ultimately leading to tissue degeneration and tendinopathy [18, 19]. In particular, resident or infiltrating immune cells (mast cells and macrophages) were shown to educate tenocytes located in the damaged tissue toward an inflammatory phenotype [18]. Fibroblasts of the damaged tendon microenvironment have a memory of inflammation up to 4 years after treatment, showing a phenotype more predisposed to the release of proinflammatory cytokines also after the cessation of the inflammatory stimulus [15]. It has been reported that immune cell-derived interleukin (IL)-1*β* prompts human tenocytes to produce inflammation mediators, such as IL-6 and IL-8, MMPs, TNF-*α* (tumor necrosis factor-*α*), COX-2 (cyclooxygenase-2), and PGE2 (prostaglandin E2) [14]. Treatment of human tendon stem cells with IL-1*β* was found to be associated with the downregulation of tendon-related genes as well as of collagen and proteoglycans, resulting in the impairment of stem cell differentiation and inhibition of tendon repair capacity. Interestingly, IL-1*β* was also found to activate the PI3K/Akt signaling pathway leading to the activation/nuclear translocation of NF-*κ*B, further supporting the key role of this transcription factor as an effective molecular target for the treatment of tendinopathies [20, 21, 22].

Another essential factor involved in the pathophysiology of tendinopathy is the extracellular matrix remodeling process that the tendon undergoes following an injury, in which MMPs are the protagonists. Physiologically, MMP action is regulated by a balanced ratio of MMPs and their inhibitors, TIMPs (tissue inhibitors of metalloproteinases). In case of tendon injury, however, inflammation causes an increase in the synthesis of MMPs, especially MMP-2 during the acute phase, which becomes responsible for the degradation of the matrix and collagen [23, 24].

Several *in vitro* studies support that, in addition to inflammation, oxidative stress plays a key role in the pathogenesis of tendinopathy, by inducing oxidative damage [25, 26]. Accordingly, in a preclinical rat model of patellar tendon injury, Fu et al. [27] observed that subcutaneous injections of hydrogen peroxide (H_2_O_2_, the most common radical oxygen species, ROS) over patellar tendons impair tendon healing and cause structural tendinopathic changes.

ROS are also produced in mitochondria as a part of the cellular respiratory process and ROS overproduction is associated with peculiar structural and functional alterations at the mitochondrial level (damage of the respiratory chain, mutations of mitochondrial DNA, alterations of the membrane permeability, and endoplasmic reticulum-derived Ca^2+^ overload), ultimately leading to cell death. In tendon cells, it has been proposed that ROS overproduction and consequent mitochondrial dysfunction are triggered by oxidative damage and play a key role in the development of tendinopathy [28, 29]. High ROS levels were reported to increase MMPs, IL-6 and PGE2 expression/secretion, and to suppress the activity of the TGF-*β*/Smad pathway, thus triggering a peculiar collagen fiber reorganization [30, 31]. Recently, it has been demonstrated that activation of mitochondrial ALDH2 (aldehyde dehydrogenase 2), the enzyme involved in the detoxification of cytotoxic aldehydes (oxidative stress-inducers), exerts cytoprotective effects in cellular and mouse models of Achilles tendinopathy [32]. Based on these observations, antioxidants, helping to prevent or counteract the effects of excessive ROS activity and oxidative stress conditions are considered promising and effective strategies for the prevention/treatment of tendon-related pathologies.

The available preventive and curative strategies for tendinopathy are still limited [33, 34]. Rehabilitation interventions are specifically aimed at reducing pain while promoting tendon healing and restoring tendon capacity, muscle contraction and patients' movement capabilities [35, 36, 37]. Surgical procedures are presently based on minimally invasive approaches [38] and innovative tissue engineering techniques (such as electrospinning, three-dimensional printing and soft lithography) have been recently developed [39, 40]. Pharmacological strategies include NSAIDs (nonsteroidal anti-inflammatory drugs), corticosteroids, platelet-rich plasma, prolotherapy, botulin toxin, hyaluronic acid, and polidocanol [41, 42, 43]. Among these, surely the most widely used approach concerns NSAIDs, although the consumption of these drugs is associated with numerous adverse events, including the inhibition of proteoglycan synthesis and cell proliferation [21]. Unfortunately, the outcomes of these interventions are still below expectations, and therefore, safer and more selective therapeutic approaches to tendon diseases are being sought.

More recently, oral supplementations of natural bioactive compounds were proposed as effective preventive (“teno-protection”), or even therapeutic, strategies for tendinopathy [44, 45, 46, 47], even if the clinical evidence is not yet sufficient to recommend the use of these products in the clinical guidelines.

As a potent antioxidant, vitamin C (Vit C) has been proposed as a potential preventive/therapeutic intervention in several oxidative stress-related diseases [48, 49, 50]. Its beneficial effects in musculoskeletal pathologies are also recognized [51, 52, 53]. *In vitro* studies demonstrated that Vit C increases collagen synthesis in cultured human tenocytes [53, 54, 55]. In line with these observations, Ömeroğlu et al. [56] reported that Vit C supplementation stimulates Achilles tendon healing by increasing early angiogenesis and collagen synthesis in a rat model of tendon rupture. Local Vit C injections were shown to reduce tendon adhesion, a common complication in tendon repair, by decreasing fibrotic response, in a chicken model [57]. In a mouse tendonitis experimental model, induced by injection of collagenase type I, this vitamin was found to improve the positive effects of adipose-derived stem cells [58]. Moreover, different human trials support that Vit C supplementation represents a potential useful strategy for tendinopathy recovery, based on its ability to increase collagen synthesis by human tenocytes [59].

Collagen is a protein characterized by a triple-helix structure that can be easily hydrolyzed enzymatically by collagenases into smaller peptides that are rapidly absorbed in the digestive tract [60]; bioactive collagen peptides, made from bovine, marine, and porcine sources [61] are present in different dietary supplements and their potential health benefits are under investigation [47, 62, 63]. In a randomized, double-blinded and placebo-controlled study in athletes with chronic ankle instability, it has been reported that a collagen peptide supplementation significantly improves ankle stability and function [64]. Similarly, it was shown that an oral supplementation of collagen peptides, in combination with calf-strengthening exercises, decreases the pain while increasing the functions, in patients suffering from Achilles tendinopathy [65]. Moreover, *in vitro* studies performed in adult mouse tenocyte lines demonstrated that the collagen-derived dipeptide prolyl-hydroxyproline improves cell homeostasis and motility [66]. The overall positive effects of collagen peptide supplementations on extracellular matrix connective tissues and recovery from injuries have been recently summarized in a comprehensive review article by Khatri et al. [67]. Importantly, the recent double-blind randomized controlled JUMPFOOD clinical study by van Dam et al. [68] pointed out an additional effect of Vit C and hydrolyzed collagen in combination with progressive tendon loading exercises for athletes with patellar tendinopathy. However, besides these promising observations, current clinical guidelines still do not support the preventive/therapeutic use of collagen peptides as food supplements.

Resveratrol (*trans*-3,5,4′-trihydroxystilbene), is a member of the stilbene family, known as phytoalexin, found in grapes and red wine, widely demonstrated to be endowed with different healthy effects, such as anti-inflammatory, antioxidant, immune-modulatory, antidiabetic, cardio- and neuroprotective, and antiaging [69, 70, 71, 72, 73]. In streptozocin-induced diabetic rats subjected to bilateral tenorrhaphy of the Achilles tendon, intraperitoneal resveratrol injections were shown to improve the process of tendon healing by increasing collagen synthesis in the repair zones [74]. Busch et al. reported that, in human tenocytes, resveratrol reverses IL-1*β*-induced apoptosis as well as PI3K and NF-*κ*B activation, while decreasing the expression of genes involved in inflammatory processes. In these cells, it also promotes the synthesis of components of the extracellular matrix, such as collagen type I and III, tenomodulin, and the tenocyte transcription factor scleraxis [21, 75]. However, the experimental data so far available assessing the anti-inflammatory activity of resveratrol in human tenocytes are still very limited. Although several clinical trials have revealed significant health promoting properties of resveratrol-based food supplements, clinical studies supporting its potential efficacy for the prevention/management of tendinopathy are still lacking.

Astaxanthin is a natural carotenoid synthesized in different marine organisms, specifically in the green microalga *H*. (*Haematococcus*) *pluvialis*, the only microorganism authorized for production of astaxanthin in EU [76, 77]. In the last years, results from *in vitro*, preclinical and clinical studies consistently pointed out that this carotenoid is endowed with different health benefits in aging as well as in a wide range of pathologies, such as cardiovascular, neurodegenerative, hepatic, renal, skin, and gastrointestinal diseases, based on its anti-inflammatory and antioxidant properties [78, 79, 80, 81, 82]. It has been also shown to protect muscle and bone health, by decreasing oxidative stress-related myoblast cell death (apoptosis) and bone resorption [83, 84]. Interestingly, in rabbit undergoing Achilles tendon injury, the use of astaxanthin has proven effective in promoting the healing process. In fact, animals treated with astaxanthin showed an increase in tendon tensive strength and a reduction in the type III/I collagen ratio, in favor of thicker and more ordered type I collagen fibers [85]. On the other hand, no clinical trials addressing the potential beneficial activity of astaxanthin as a preventive or treatment strategy for tendinopathy have been so far carried out.

Although each of these natural bioactive compounds has been studied alone, their possible synergistic activity has never been investigated. Here we evaluated the efficacy and the synergistic effects of a combination of Vit C, bioactive collagen peptides, resveratrol and astaxanthin (Mix) in counteracting the proinflammatory and prooxidant activity of IL-1*β* and H_2_O_2_, respectively, in human primary tenocytes. The aim of this study was therefore to characterize the mechanism of action of the single compounds and their Mix on cellular markers that generally underlie tendinopathies.

## 2. Materials and Methods

### 2.1. Compounds and Mix Preparation

Vit C (L-Ascorbic acid), interleukin-1*β* (IL-1*β*), and hydrogen peroxide (H_2_O_2_) were purchased from Sigma–Aldrich (St. Louis, MI, USA). Bioactive collagen peptides (TENDOFORTE®; Coll) were provided by GELITA (Eberbach, Germany), trans-resveratrol (as Veri-Sperse®, combining Veri-te™ Resveratrol by Evolva (Switzerland) with LipiSperse® technology by Pharmako Biotechnologies (Australia); Resv) was supplied by Evolva, and astaxanthin (AstaReal® EL25; Asx) was donated by AstaReal® AB (Nacka, Sweden).

**Figure 1:**
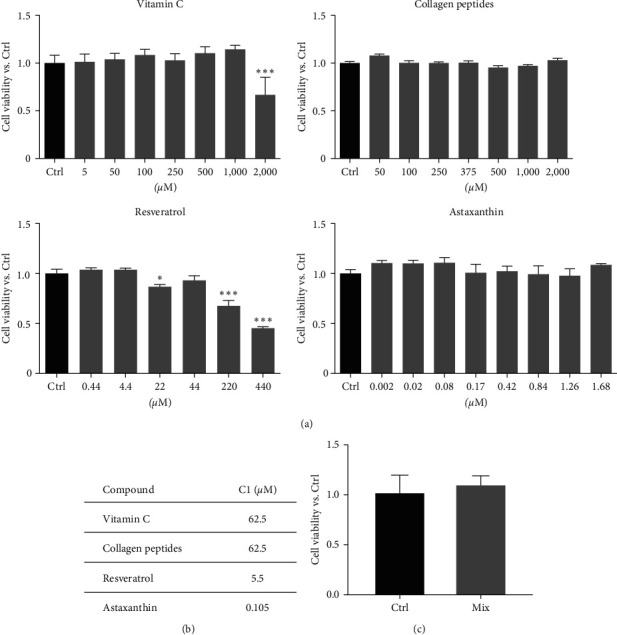
Effects of single compounds and their combination (Mix) on tenocyte viability. (a) Tenocytes were treated with Vit C (5–2,000 *µ*M), Coll (50–1,500 *µ*M), Res (0,44–440 *µ*M), and Asx (0.002–1.68 *µ*M) for 24 hr. The cell viability was assessed by MTT assay, (b) doses of each compound selected in order to obtain the most appropriate combination (Mix) to exclude cytotoxic effects. (c) Tenocytes were treated with the Mix for 24 hr and the cell viability was then assessed by MTT assay. (a) and (c) data are expressed as fold change of the cell viability vs. Ctrl (controls, untreated cells) and are presented as the mean of three separate experiments ± S.E.  ^*∗∗∗*^*p* < 0.001 and  ^*∗*^*p* < 0.05.

Vit C and Coll were diluted in water, at a stock concentration of 100 mM and 100 mg/mL, respectively; Resv and Asx were dissolved in dimethyl sulfoxide (DMSO), at a stock concentration of 50 and 100 mg/mL, respectively. Stock solutions were stored at −20°C. The mixture (Mix) was prepared by mixing appropriate amounts of the single compounds, selected as described here in Section 2.3, as well as in the Results section (Section 3.1; Figure 1(b).

### 2.2. Cell Cultures

Cryopreserved human primary tenocytes (TEN-F, Lot# TEN031320A) were purchased from a commercial supplier (Zen-Bio Inc., Durham, NC, USA). Specifically, the cells were obtained from the Achilles tendon of a male healthy donor aged 74 years, who has undergone elective surgery. Upon thawing, the cells were routinely cultured in Dulbecco Modified Eagle's Medium (DMEM), supplemented with 10% Fetal Bovine Serum (FBS), 1% L-glutamine and 1% antibiotics, in a humidified atmosphere of 5% CO_2_/95% air at 37°C, and expanded for no more than 5 passages. The cells were detached through trypsin-EDTA solution when reaching 80% confluence. For the experiments requiring an inflamed tenocyte model, cells were treated with IL-1*β* (10 ng/mL, O/N) [86, 87, 88]. For the experiments requiring an oxidative stress model, the cells were treated with H_2_O_2_ (1,5 mM for 2 hr) [55, 89, 90].

### 2.3. Cytotoxicity Assay

To assess the lack of cytotoxicity a classical MTT assay was performed as previously described [91, 92]. This assay measures the mitochondrial reductive activity and is widely utilized as an indicator of the cell metabolic activity or cell proliferation/viability/cytotoxicity [93]. Tenocytes were plated in 48-well plates (6 × 10^3^ cells/well) and after 48 hr were subjected to the appropriate treatment. Specifically, dose–response curves were performed for each compound in order to assess an eventual cytotoxic effect (Vit C: 0.88–352 *µ*g/mL, Coll: 100–3,000 *µ*g/mL, Resv: 0.1–100 *µ*g/mL, and Asx: 0.001–1 *µ*g/mL). Dosages have been chosen in a physiological range, based on pharmacokinetic data from the literature [94, 95, 96, 97, 98, 99, 100]. Moreover, for each compound, the dose to obtain the most appropriate combination (Mix) devoid of cytotoxic effects was selected for the subsequent experiments. The DMSO at final concentration of 0.1% used to dilute compounds and Mix, was also added to all experiments. After 24 hr treatment, cells were incubated with a solution of MTT (3-(4,5-dimethyltiazol-2-yl)-2,5-diphenyltetrazolium bromide, 0.5 mg/mL; Sigma–Aldrich, for 1 hr at 37°C. The violet precipitate was dissolved in isopropanol and absorbance at 550 nm was measured through an EnSpire Multimode Plate reader (PerkinElmer, Milano, Italy).

For the experiments requiring an inflamed tenocyte model, cells were treated with IL-1*β* [86, 87, 88]. To confirm the lack of toxicity of IL-1*β*, either alone or in combination with the single compounds or the Mix, tenocytes were plated for 32 hr. The cells were then challenged with IL-1*β* (10 ng/mL, O/N) followed by the treatment with the single compounds or the Mix for 24 hr. The cell viability was assessed by MTT assay.

For the experiments requiring an oxidative stress model, cells were treated with H_2_O_2_ [55, 89, 90]. To confirm the lack of toxicity of H_2_O_2_, either alone or in combination with the single agents or the Mix, tenocytes were plated for 48 hr. The cells were then pretreated, for 24 hr, with the single compounds or the Mix and subsequently challenged with H_2_O_2_ (1.5 mM for 2 hr). The cell viability was assessed by MTT assay.

### 2.4. ELISA Assay

As indicative of inflammation and tissue remodeling processes, the secreted amount of IL-6 and MMP2, respectively, were measured through the ELISA assay. Tenocytes were pretreated with 10 ng/mL IL-1*β* O/N and then with each compound, or their combination, for additional 24 hr (as described above): at this time point, the supernatant was collected and analyzed for IL-6 and MMP2 secretion, following manufacturer's protocols (DuoSet ELISA kits, R&D Systems, Minneapolis, MN, USA). The absorbance at 450 and 540 nm was measured through an EnSpire Multimode Plate reader (PerkinElmer).

To compare the anti-inflammatory activity of each compound and their combination (Mix), the synergistic effect of the four compounds was calculated as previously described by Ranjbar Nedamani et al. [101].

### 2.5. Immunofluorescence

NF-*κ*B nuclear translocation was assessed through immunofluorescence staining. Tenocytes were seeded on polylysine-coated 13-mm coverslips (3 × 10^4^ cells/dish), in 24-well plates for 48 hr and treated with: IL-1*β* (10 ng/mL) for 1 hr; Mix for 24 hr; Mix for 24 hr followed by IL-1*β* (10 ng/mL) for 1 hr [102]. Then, the cells were fixed with a 4% PFA/2% saccharose (W/v) solution and stained with the NF-*κ*B primary antibody (Cell Signaling Technology, Danvers, MA, USA) for 4 hr, followed by an anti-rabbit-Alexa488 secondary antibody for 1 hr, and nuclei were counterstained with DAPI (Sigma–Aldrich) for 15 min. The labeled cells were observed under a Zeiss Axiovert 200 microscope with a 32 × 1.4 objective lens linked to a Coolsnap Es CCD camera (Roper Scientific-Crisel Instruments, Roma, Italy).

### 2.6. ROS Production

As indicative of oxidative stress, ROS production was assessed by means of a fluorescence-based assay [103, 104, 105, 106]. Tenocytes were pretreated with each compound or with Mix for 24 hr (as described above). The treatment was removed, the probe H_2_DCFDA 10 *μ*M (2′, 7′-dichlorodihydrofluorescein diacetate, ThermoFisher Scientific, Waltham, MA, USA) was added to the culture for 1 hr and then the cells were treated with H_2_O_2_ (1.5 mM for 2 hr) [55, 89, 90]. The ROS-mediated conversion of H_2_DCFDA to the fluorescent molecule DCF (2′, 7′-dichlorofluorescein, *λ*_em_ 517–527 nm) was measured through an EnSpire Multimode Plate reader (PerkinElmer). Basal fluorescence from control wells was removed after data acquisition.

### 2.7. Statistical Analysis

The values of fold change (the ratio between treated values and respective control values, one-fold increase) were compared among Mix and the components administered in single through a model of analysis of variance (ANOVA) including treatment (Mix and single components), activation (presence/absence of IL-1*β* or H_2_O_2_) and their interaction as predictors. The fold change was the dependent variable. Each experimental group consisted of at least three replicates and each experiment was repeated three times. The means of the three replicates of each independent experiment, yielding a total of three independent measures, were then statistically analyzed. All analyses were done in R 3.6.1 (R Core Team 2019). Otherwise stated, data reported are means and standard errors (R Core Team. 2019. R: A Language and Environment for Statistical Computing. Vienna, Austria: R Foundation for Statistical Computing. Available from: http://www.R-project.org/). Images were performed with GraphPad Prsim5 (GraphPad Software, San Diego, CA, USA). A *p* value < 0.05 was considered statistically significant.

## 3. Results

### 3.1. Effects of Single Compounds and Their Combination (Mix) on Tenocyte Viability

Experiments were performed to assess the effects of the single compounds (Vit C, Coll, Resv, and Asx) and their combination (Mix) on human tenocyte viability. First, the cells were treated with Vit C (5–2,000 *µ*M), Coll (50–1,500 *µ*M), Resv (0.44–440 *µ*M), and Asx (0.0002–1.68 *µ*M) for 24 hr. The effects of the treatment on cell viability were assessed by MTT assay.  Figure 1(a) shows that Vit C did not affect the cell viability at the dose range of 5–1,000 *µ*M, while it decreased this parameter at 2000 *µ*M. Resv exerted a cytotoxic effect at the doses 22, 220, and 440 *µ*M but was devoid of any effect at the other concentrations. Finally, Coll and Asx did not affect the cell viability at any of the doses evaluated. Based on these preliminary results, the following doses of each compound were selected to obtain the most appropriate combination (Mix) to exclude cytotoxic effects: Vit C 62.5 *µ*M, Coll 62.5 *µ*M, Resv 5.5 *µ*M, and Asx 0.105 *µ*M (Figure 1(b)). Tenocytes were then treated with the Mix for 24 hr and, as expected, we could observe that the Mix did not affect the cell growth (Figure 1(c)).

### 3.2. Setting Up the Experimental Models of Tenocyte Inflammation and Oxidative Stress

**Figure 2:**
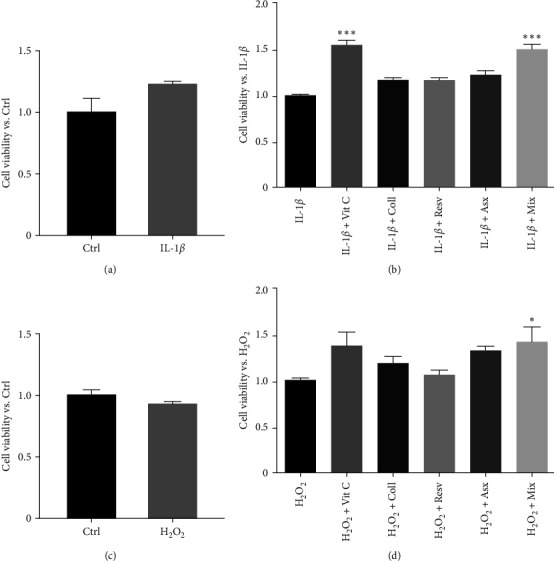
Effects of IL-1*β* and H_2_O_2_, either alone or in combination with single compounds or Mix, on tenocyte viability. (a) Tenocytes were pretreated with IL-1*β* (10 ng/mL, O/N), (b) tenocytes were pretreated with IL-1*β*, as in (a) and then with the single compounds (62.5 *µ*M Vit C, 62.5 M Coll, 5.5 *µ*M Resv, and 0.105 *µ*M Asx) or the Mix for 24 hr, (c) tenocytes were treated with H_2_O_2_ (1.5 mM) for 2 hr, and (d) tenocytes were pretreated (24 hr) with the single compounds or the Mix and then challenged with H_2_O_2_, as in (c). In all the experiments, the cell viability was assessed by MTT assay. (a) and (c) data are expressed as fold change of the cell viability vs. Ctrl (controls, untreated cells). (b) and (d) data are expressed as fold change viability vs. IL-1*β* (controls). Results are presented as the mean of three separate experiments.  ^*∗∗∗*^*p* < 0.001,  ^*∗*^*p* < 0.05.

IL-1*β* has been shown to foster inflammation in human tenocytes by stimulating the release of different inflammatory mediators, such as the cytokine IL-6 and the MMP-2 [14]. Preliminary experiments were performed to exclude cytotoxic effects of the cytokine (alone or in combination with natural compounds). First, tenocytes were treated with IL-1*β* (10 ng/mL O/N) and the cell viability was assessed by MTT assay.  Figure 2(a) shows that IL-1*β* alone does not affect tenocyte viability when compared to Ctrl (control, untreated cells). Then, the cells were treated with IL-1*β* (10 ng/mL, O/N) followed by the treatment with the single compounds (62.5 *µ*M Vit C, 62.5 *µ*M Coll, 5.5 *µ*M Resv, and 0.105 *µ*M Asx) or the Mix for 24 hr. By MTT assay, we could observe that Coll, Resv, and Asx, in combination with IL-1*β*, do not affect the cell viability when compared to the cytokine alone. Cotreatment of the cells with Vit C or Mix with IL-1*β* resulted in an increased metabolic activity in comparison to IL-1*β* alone (Figure 2(b)).

Exposure to H_2_O_2_ is a widely validated procedure to boost oxidative damage/stress in different cell types.

Preliminary experiments were performed to exclude a cytotoxic activity of the selected dose of H_2_O_2_, either alone or in combination with the natural compounds. First, tenocytes were treated with H_2_O_2_ (1.5 mM) alone for 2 hr; by MTT assay we confirmed that H_2_O_2_ does not affect the cell viability in comparison to Ctrl (control, untreated cells; Figure 2(c)). Then, tenocytes were challenged with the single compounds (62.5 *µ*M Vit C, 62.5 *µ*M Coll, 5.5 *µ*M Resv, and 0.105 *µ*M Asx) or the Mix for 24 hr followed by the treatment with H_2_O_2_ (1.5 mM) for 2 hr.  Figure 2(d) shows that the combinations of H_2_O_2_ with Vit C, Coll, Resv, or Asx do not affect the cell viability when compared to controls (H_2_O_2_). The combination of H_2_O_2_ with Mix significantly, although slightly, increased the metabolic activity with respect to the H_2_O_2_ alone (Figure 2(d)).

Based on the main aims of these preliminary experiments, we can conclude that these data support a lack of cytotoxicity of both IL-1*β* and H_2_O_2_ when given alone or in combination with the single compounds or the Mix.

**Figure 3:**
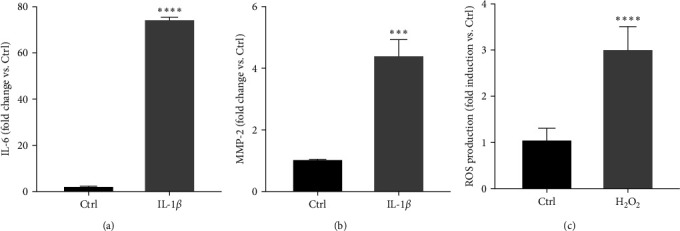
Setting up the experimental models of tenocyte inflammation and oxidative stress. (a) and (b) Tenocytes were treated with IL-1*β* (10 ng/mL, O/N). The levels of IL-6 and MMP-2, markers of inflammation, were then evaluated in the culture media by the Elisa assay, and (c) tenocytes were treated with H_2_O_2_ (1.5 mM, 2 hr) and the levels of ROS, and oxidative stress markers, were detected by fluorescence-based assay. Data are expressed as fold change vs. Ctrl (controls, untreated cells) and are presented as the mean of three separate experiments ± S.E.  ^*∗∗∗∗*^*p* < 0.0001 and  ^*∗∗∗*^*p* < 0.001.

To set up the appropriate experimental model to mimic *in vitro* cellular inflammation, tenocytes were treated with IL-1*β* (10 ng/mL, O/N) [86]; IL-6 and MMP-2 levels were then evaluated in the culture media by the Elisa assay and compared to the levels observed in Ctrl (control, untreated cells). Figures 3(a) and 3(b) show that IL-1*β* significantly increased the secretion levels of both inflammatory mediators (IL-6 *p* < 0.0001; MMP-2 *p* < 0.001).

To establish the experimental model of cellular oxidative stress in human tenocytes, the cells were treated with H_2_O_2_ (1.5 mM, 2 hr) and ROS levels were detected by fluorescence-based assay and compared to the levels observed in Ctrl (control, untreated cells). As expected, we could observe that ROS production was significantly increased after exposure to H_2_O_2_ (*p* < 0.0001; Figure 3(c)).

### 3.3. Effects of Single Compounds and Mix on IL-1*β*-Induced IL-6 Secretion

**Figure 4:**
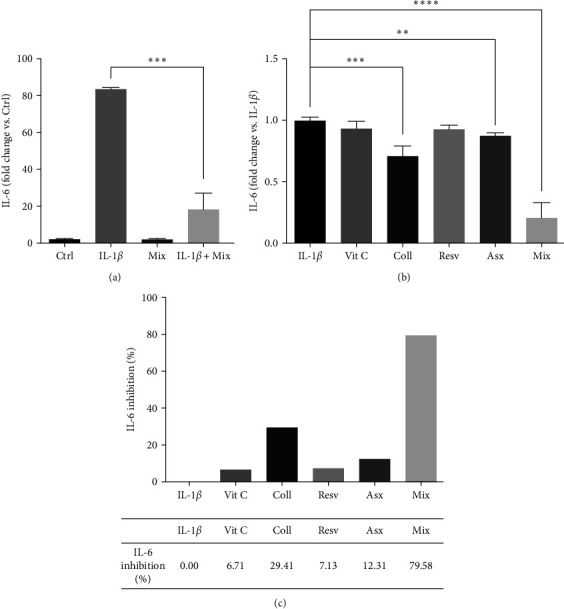
Effects of single compounds and Mix on IL-1*β*-induced IL-6 secretion. (a) First, we evaluated the effects of the Mix on IL-6 secretion in Ctrl (control, untreated cells) and in IL-1*β*-treated (IL-1*β*) cells. Tenocytes were untreated or treated with IL-1*β* (10 ng/mL, O/N) and then with the Mix for additional 24 hr. IL-6 levels in the culture media were evaluated by the ELISA assay. Data are expressed as fold change vs. Ctrl (controls, untreated cells) and are presented as the mean of three separate experiments ± S.E., (b) tenocytes were treated with IL-1*β*, as in (a), and then with each compound or the Mix for additional 24 hr. IL-6 levels in the culture media were evaluated by the ELISA assay. Data are expressed as fold change vs. IL-1*β* (controls + IL-1*β*) and are presented as the mean of three separate experiments ± S.E., and (c) expression of the results reported in (b) as the percentage of IL-6 inhibition of the four compounds in combination (Mix) compared to that of their separate effect at the same doses.  ^*∗∗∗∗*^*p* < 0.0001,  ^*∗∗∗*^*p* < 0.001 and  ^*∗∗*^*p* < 0.01.

First, we evaluated the effects of the compound combination (Mix) on IL-1*β*-induced IL-6 secretion. Human tenocytes were treated with 10 ng/mL IL-1*β* O/N and then with the Mix for additional 24 hr.  Figure 4(a) shows that Mix did not affect IL-6 secretion from noninflamed, Ctrl (control, untreated cells), while it significantly decreased its release from IL-1*β* treated cells (IL-1*β* + Mix) (*p* < 0.001).

Then, we compared the anti-inflammatory effects of the Mix with those of each individual compound. Tenocytes were pretreated with IL-1*β*, as above, and then with the single compounds (62.5 *µ*M Vit C, 62.5 *µ*M Coll, 5.5 *µ*M Resv, and 0.105 *µ*M Asx) or the Mix for 24 hr; the cells pretreated with IL-1*β* were used as control (IL-1 *β*). As shown in Figure 4(b), and in accordance with what is shown in Figure 4(a), Mix significantly decreased IL-6 secretion when compared to IL-1*β* treated cells (*p* < 0.0001). Among the single compounds, only Coll and Asx were able to significantly counteract IL-1*β*-induced IL-6 secretion (Coll vs. IL-1*βp* < 0.001; Asx vs. IL-1*βp*  < 0.01). Comparing the activity of the Mix with the activity of each single compound on the expression of IL-6, we observed that the efficacy of the Mix in reducing IL-6 secretion is statistically greater than each of the single ingredients (Mix vs. Vit C, Coll, Resv, and Asx *p* < 0.001; Figure 4(b)). Notably, by measuring the percentage of inhibition of IL-6 release induced by the single compounds and Mix compared to the inflamed control, we could observe a synergistic effect of the four compounds in decreasing IL-6 release, as their combined effect was greater than the sum of each single compound's activity (as evaluated by analyzing the percentage of IL-6 inhibition compared to IL-1*β* cells) [101, 107, 108, 109]. In fact, relative to IL-*β* (the control), the Mix reduced IL-6 production by about 80% while the sum of the inhibitory effects of the single compounds was about 55% relative to IL-*β* (Figure 4(c)).

### 3.4. Effects of Mix on IL-1*β*-Induced NF-*κ*B Translocation

**Figure 5:**
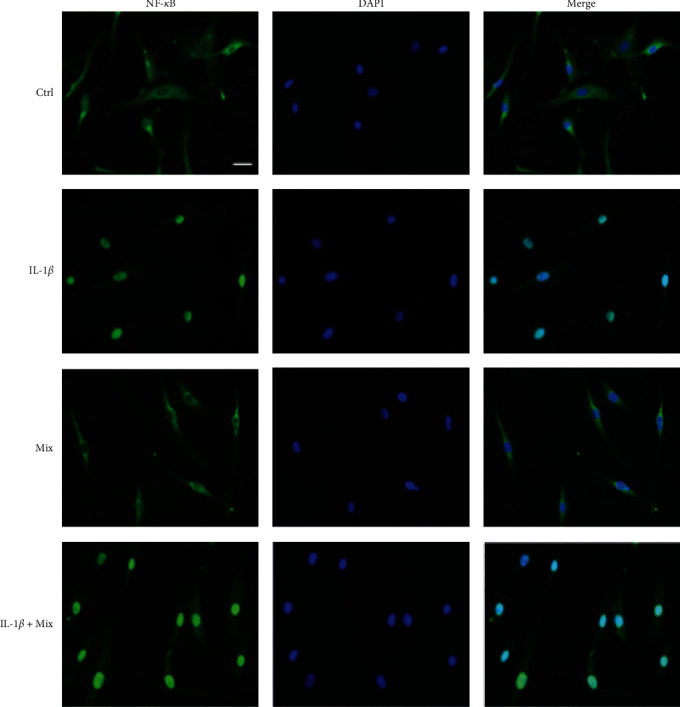
Effects of the Mix on IL-1*β*-induced nuclear translocation of NF-*κ*B. Tenocytes were treated with IL-1*β* (10 ng/mL) for 1 hr; Mix for 24 hr; and Mix for 24 hr followed by IL-1*β* for 1 hr. Intracellular NF-*κ*B localization was evaluated by immunofluorescence staining. Ctrl (controls, untreated cells). Scale bar, 20 *µ*m.

The inflammatory effects of IL-1*β* are well known to be mediated by the MAPK and MYD88/TRAF6/TAK1/IKKl (leading to phosphorylation, ubiquitination, and degradation of I*κ*B-*α* pathway) signaling cascades, allowing NF-*κ*B translocation to the nucleus, further supporting the key role of this transcription factor as an effective molecular target for the treatment of tendinopathies [20, 21, 110]. To confirm the anti-inflammatory activity of the compound combination, tenocytes were pretreated with IL-1*β* (10 ng/mL) for 1 hr; Mix for 24 hr; and Mix for 24 hr followed by IL-1*β* (10 ng/mL) for 1 hr. Intracellular NF-*κ*B localization was analyzed by immunofluorescence staining.  Figure 5 shows that, as expected, IL-1*β* triggered NF-*κ*B translocation into the nuclei. On the contrary, treatment with the Mix, without inducing inflammation, did not involve the translocation of NF-*κ*B, whose signal remained at the cytoplasmic level, as in the Ctrl (control, untreated cells). By pretreating the cells with Mix and inducing inflammation, a part of NF-*κ*B translocated into the nucleus, while a part remained at the cytoplasmic level.

### 3.5. Effects of Single Compounds and Mix on IL-1*β*-Induced MMP-2 Secretion

**Figure 6:**
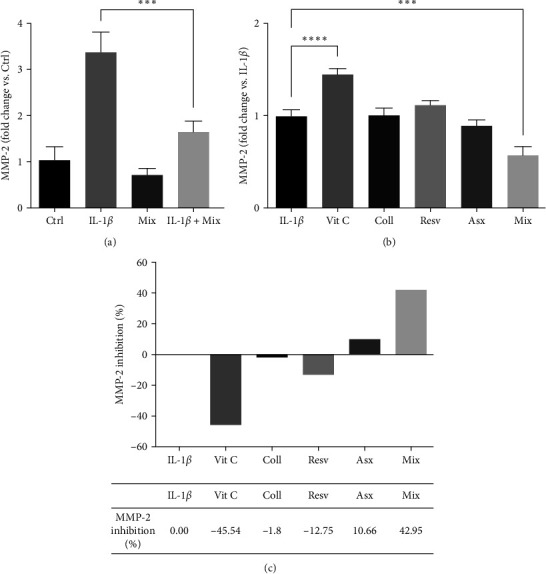
Effects of single compounds and Mix on IL-1*β*-induced MMP-2 secretion. (a) First, we evaluated the effects of the Mix on MMP-2 secretion in Ctrl (control, untreated cells) and in IL-1*β*-treated (IL-1*β*) cells. Tenocytes were untreated or treated with IL-1*β* (10 ng/mL, O/N) and then with the Mix for additional 24 hr. MMP-2 levels in the culture media were evaluated by the ELISA assay. Data are expressed as fold change vs. Ctrl (controls, untreated cells) and are presented as the mean of three separate experiments ± S.E., (b) tenocytes were treated with IL-1 *β*, as in (a), and then with each compound or the Mix for additional 24 hr. MMP-2 levels in the culture media were evaluated by the ELISA assay. Data are expressed as fold change vs. IL-1*β* (controls + IL-1*β*) and are presented as the mean of three separate experiments ± S.E.  ^*∗∗∗∗*^*p* < 0.0001,  ^*∗∗∗*^*p* < 0.001, and (c) expression of the results reported in (b) as the percentage of MMP-2 inhibition of the four compounds in combination (Mix) compared to that of their separate effect at the same doses.

In addition to IL-6, the inflammatory activity of IL-1*β* in tenocytes was reported to be mediated by different factors such as MMP-2, an enzyme deeply involved in extracellular matrix degradation and remodeling [14]. We first investigated the effects of the Mix on IL-1*β*-induced MMP-2 secretion. Human tenocytes were treated with 10 ng/mL IL-1*β* O/N and then with the Mix for additional 24 hr.  Figure 6(a) shows that Mix did not affect MMP-2 secretion from noninflamed, Ctrl (control, untreated cells), while it significantly decreased its release from inflamed tenocytes (IL-1*β* + Mix; *p* < 0.001).

Then, we compared the effects of the Mix with those of each compound. Human tenocytes were pretreated with IL-1*β* (as above) and then with the single compounds (62.5 *µ*M Vit C, 62.55 *µ*M Coll, 5.5 *µ*M Resv, and 0.105 *µ*M Asx) or the Mix for additional 24 hr. IL-1*β* pretreated cells were used as control (IL-1*β*). Among the individual compounds, Coll, Resv and Asx did not affect IL-1*β*-induced MMP-2 secretion, while Vit C significantly stimulated its release (*p* < 0, 0001). The data obtained on the activity of Vit C indicates that it exerts a negative effect on the matrix remodeling, while the other active ingredients do not bring any benefit. On the contrary, treatment of tenocytes with Mix significantly counteracted IL-1*β*-induced MMP-2 secretion when compared to control cells (IL-1*β;p* < 0.001). In particular, the Mix showed its efficacy in reducing MMP-2 expression when compared to single compound administration (Mix vs. Vit C *p* < 0.001; Mix vs. Coll *p* < 0.001; Mix vs. Resv *p* < 0.001; and Mix vs. Asx *p* < 0.01; Figure 6(b)).

By measuring the percentage of inhibition of MMP-2 release induced by the single compounds and Mix compared to the inflamed control we could observe a synergistic effect of the four compounds in hampering MMP-2 release since their combined effect was greater than the sum of each individual compound's activity (as evaluated by measuring the percentage of MMP-2 inhibition compared to IL-1*β* cells) [107]. The Mix, indeed, was able to reduce the release of MMP-2 by about 43%, compared to control (Figure 6(c)).

### 3.6. Effects of Single Compounds and Mix on H_2_O_2_-Induced ROS Production

**Figure 7:**
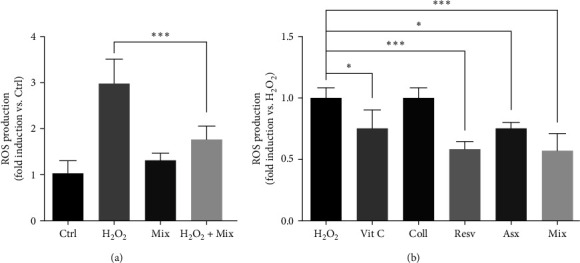
Effects of single compounds and Mix on H_2_O_2_-induced ROS production. (a) First, we assessed the effects of the Mix on ROS production in Ctrl (control, untreated cells) and in H_2_O_2_-treated cells. Tenocytes were treated with the Mix for 24 hr and then with H_2_O_2_ (1.5 mM, 2 hr). ROS levels in the culture media were measured by fluorescence analysis after staining with 2′,7′-dichlorofluorescin diacetate. Data are expressed as fold change vs. Ctrl (controls, untreated cells) and are presented as the mean of three separate experiments ± S.E., and (b) tenocytes were treated with single compounds or the Mix, followed by H_2_O_2_, as described in (a). ROS levels in the culture media were measured by fluorescence analysis after staining with 2′,7′-dichlorofluorescin diacetate. Data are expressed as fold change vs. H_2_O_2_ (controls + H_2_O_2_) and are presented as the mean of three separate experiments ± S.E.  ^*∗∗∗*^*p* < 0.001 and  ^*∗*^*p* < 0.05.

It is well established that oxidative stress plays a key role in the development of tendinopathy [28, 29]. Here, we first investigated the effects of the Mix on H_2_O_2_-induced ROS production by means of a fluorescence-based assay. As shown in Figure 7(a), Mix did not affect ROS production from Ctrl (control, untreated cells), while it significantly counteracted their production from H_2_O_2_-stimulated tenocytes (H_2_O_2_ + Mix; *p* < 0.001).

Then, we compared the effects of the Mix with those of each compound. Tenocytes were pretreated with the single compounds (62.5 *µ*M Vit C, 62.5 *µ*M Coll, 5.5 *µ*M Resv, and 0.105 *µ*M Asx) or with the Mix for 24 hr, and then challenged with H_2_O_2_ (1.5 mM) for 2 hr.  Figure 7(b) shows that Resv, Asx, and Vit C significantly decreased ROS levels (Resv vs. H_2_O_2_*p* < 0.001; Asx vs. H_2_O_2_*p* < 0.05; and Vit C vs. H_2_O_2_*p* < 0.05). Mix was also found to be able to inhibit the production of ROS (with an efficacy of 42% vs. H_2_O_2_) although its activity was observed to be comparable to that of the single active ingredients, apart from collagen.

## 4. Discussion

Tendinopathy is a musculoskeletal disease commonly affecting general population with different incidence and prevalence based on the tendon involved, and intrinsic and extrinsic factors as age, sex, comorbidities, physical activity, sports training, and occupational tasks [4]. The pathophysiology involves first of all inflammation and oxidative stress, which in turn determine extracellular matrix remodeling.

Different strategies, including rehabilitation interventions, surgical procedures, and tissue engineering approaches, were developed to prevent and treat tendinopathy [33]. However, the outcomes of these strategies are still limited. Very often these therapies look at the resolution of the symptoms, but they have no value in restoring the correct tendon structure. Treatment of tendinopathies is still a challenge, mostly because a large portion of patients do not show a complete recovery from the injury [4]. Recently, natural compounds have been proposed as potential preventive or therapeutic interventions for this disease, thanks to their ability to scavenge free radicals and reduce the damage caused by inflammation [44, 45, 46].

The aim of this study was to assess the effects of a combination of biomolecules contained in the Mix (Vit C, bioactive collagen peptides, resveratrol, and astaxanthin) in counteracting proinflammatory and oxidative stress stimuli (IL-1*β* and H_2_O_2_, respectively) in human tenocytes. The rationale of this experimental approach is based on the observation that it would be necessary to consider this pathology by looking at the complexity of its pathophysiology and thus simultaneously to target both inflammation and oxidative stress, supporting the tissue remodeling necessary to restore the proper function of tendons, with a view to prevent recurrence.

First, we performed experiments to select the right concentration of each compound to assess their anti-inflammatory and antioxidant effects without affecting tenocyte viability: Vit C, 62.5 *µ*M; Coll, 62.5 *µ*M; Resv, 5.5 *µ*M; and Asx, 0.105 *µ*M. Once selected the appropriate concentrations of each compound to be used in their mixture, we analyzed the effects of the single biomolecules or their Mix on IL-1*β*-induced inflammatory mediators. We found that among the single compounds, only Coll and Asx were able to significantly reduce IL-6 secretion; on the other hand, Mix significantly decreased the release of this cytokine, being significantly more effective than all single compounds. Importantly, these results highlighted a synergistic activity of the combination of the four compounds in reducing inflammation. In line with these data, we also observed that treatment of tenocytes with the Mix counteracts IL-1*β*-induced nuclear translocation (*i.e*., activation) of the transcription factor NF-*κ*B.

This important result could highlight the mechanism of action of the anti-inflammatory activity exerted by the Mix. By preventing the translocation of NF-*κ*B in the nucleus upstream, the Mix is actually able to significantly reduce the NF-*κ*B pathway downstream, as demonstrated by the synergistic action on the production of IL-6.

This result is further confirmed if another aspect of tendinopathies is taken into consideration. In case of tendon disorders, there is an increase in the production of MMPs, in particular of MMP-2 during the acute phase, responsible for the degradation of the extracellular matrix and collagen [23].

*In vitro*, the proinflammatory activity of IL-1*β* is known to induce MMP-2 release. Here, we demonstrated that Coll, Resv, and Asx did not affect IL-1*β*-induced MMP-2 secretion; indeed, according to what has been demonstrated in a recent study [53], Vit C showed a stimulating activity. Importantly, treatment of the cells with Mix significantly hampered IL-1 *β*-triggered MMP-2 secretion from human tenocytes. These results support a synergistic effect of the four compounds in reverting MMP-2 release since their combined effect was greater than the sum of each individual compound's activity.

These results indicate that the Mix is able, on one hand, to decrease the inflammation of tenocytes, and, on the other hand, demonstrate its potential in preserving the correct structure of tendons.

Oxidative stress is another key molecular mechanism underlying the development of tendinopathy. Here, we analyzed the effects of the single compounds and their Mix on H_2_O_2_-induced ROS production in human tenocytes. We could observe that Vit C, Resv, and Asx significantly decreased ROS levels. Treatment with the Mix also significantly counteracted H_2_O_2_-induced ROS overproduction although a synergistic effect could not be observed.

**Figure 8:**
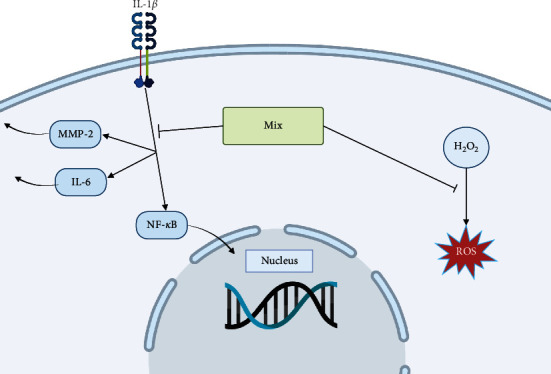
Schematic representation of the anti-inflammatory and antioxidant properties of Mix (vitamin C, collagen peptides, resveratrol, and astaxanthin) in human tenocytes. The Mix inhibits IL-1*β*-induced IL-6 and MMP-2 secretion and NF-*κ*B nuclear translocation. Mix also counteracts H_2_O_2_-triggered ROS production.

Taken together, these results strongly support that a combination of Vit C, Coll, Resv, and Asx significantly (and more effectively than the single compounds) counteracts IL-1*β*-induced inflammation; the combination also significantly impairs H_2_O_2_-triggered oxidative stress. Considering these results in their complexity, we can affirm that only the combination of these compounds demonstrates the ability to deal with all aspects of tendinopathies at the same time. For example, Coll and Asx are able to decrease the IL-6 production, but they have no effect on the release of MMP-2. Concerning ROS production, Coll has no effect, while Asx is able to reduce it. Resv and Vit C, on the other hand, demonstrated antioxidant power, while they did not give rise to an anti-inflammatory response. From all this, it emerges that the Mix proved to be able to: (i) exert an anti-inflammatory activity, demonstrated by inhibiting both the release of IL-6 and the nuclear translocation of NF-*κ*B; (ii) inhibit the release of MMP-2, assuming an action on the remodeling of the tendon cell matrix; and (iii) exert an antioxidant activity (Figure 8).

To the authors' knowledge, the relevance and innovation of these data, is based on the observation that the anti-inflammatory and antioxidant activity of a combination of Vit C, Coll, Resv, and Asx in human tenocytes, as well as in other models of chronic diseases, has never been investigated so far.

Noteworthy, these data are in line with recent evidence strongly supporting that supplementation of natural compound combinations can represent novel effective strategies for both the prevention and treatment (*i.e*., as an adjunctive method to standard therapies) of various inflammation- and oxidative stress-related diseases [111, 112, 113, 114, 115, 116, 117, 118]. Notably, in light of our results, the advantage of using a Mix-based supplement could be that of acting on the molecular mechanisms underlying the onset of tendinopathies.

## 5. Conclusion

The impact of dietary supplements, based on the combination of different natural compounds, on the prevention and treatment of tendinopathy in a general human population has been recently discussed in a review article by Hijlkema et al. [46]. The authors underline that some studies showed promising clinical implications for the use of dietary supplements, particularly those containing collagen-derived peptides. However, limited scientific quality and variety among the different studies on nutrient intake and tendon health in tendinopathy make it impossible to draw definitive conclusions and recommendations on dietary supplement intake, for either the prevention or the treatment of tendinopathy [46].

Our study points out that a new mixture of natural compounds, based on the combination of Vit C, Coll, Resv, and Asx, significantly impairs proinflammatory stimuli and oxidative stress damage in human tenocytes and has promising properties for future application in tendinitis.

## Data Availability

The data used to support the findings of this study are included in the manuscript.
